# Immunoproteasome Inhibition Ameliorates Aged Dystrophic Mouse Muscle Environment

**DOI:** 10.3390/ijms232314657

**Published:** 2022-11-24

**Authors:** Luana Tripodi, Davide Molinaro, Francesco Fortunato, Carolina Mella, Barbara Cassani, Yvan Torrente, Andrea Farini

**Affiliations:** 1 Stem Cell Laboratory, Dino Ferrari Center, Department of Pathophysiology and Transplantation, University of Milan, 20122 Milan, Italy; 2 Department of Pathophysiology and Transplantation, Università degli Studi di Milano, 20122 Milan, Italy; 3 Department of Medical Biotechnologies and Translational Medicine, Università Degli Studi di Milano, 20089 Milan, Italy; 4 Humanitas Clinical and Research Center IRCCS, Rozzano, 20089 Milan, Italy; 5 Neurology Unit, Fondazione IRCCS Ca’ Granda Ospedale Maggiore Policlinico, 20122 Milan, Italy

**Keywords:** immunoproteasome, muscle mass, inflammation, sarcopenia, aging

## Abstract

Muscle wasting is a major pathological feature observed in Duchenne muscular dystrophy (DMD) and is the result of the concerted effects of inflammation, oxidative stress and cell senescence. The inducible form of proteasome, or immunoproteasome (IP), is involved in all the above mentioned processes, regulating antigen presentation, cytokine production and immune cell response. IP inhibition has been previously shown to dampen the altered molecular, histological and functional features of 3-month-old mdx mice, the animal model for DMD. In this study, we described the role of ONX-0914, a selective inhibitor of the PSMB8 subunit of immunoproteasome, in ameliorating the pathological traits that could promote muscle wasting progression in older, 9-month-old mdx mice. ONX-0914 reduces the number of macrophages and effector memory T cells in muscle and spleen, while increasing the number of regulatory T cells. It modulates inflammatory markers both in skeletal and cardiac muscle, possibly counteracting heart remodeling and hypertrophy. Moreover, it buffers oxidative stress by improving mitochondrial efficiency. These changes ultimately lead to a marked decrease of fibrosis and, potentially, to more controlled myofiber degeneration/regeneration cycles. Therefore, ONX-0914 is a promising molecule that may slow down muscle mass loss, with relatively low side effects, in dystrophic patients with moderate to advanced disease.

## 1. Introduction

Duchenne muscular dystrophy (DMD) is a fatal disease caused by mutations in the dystrophin gene. In DMD, inflammation and muscle invasion by several immune cells are triggered by damage-associated molecular patterns (DAMPs) released by injured myofibers, oxidative stress and defective calcium handling, and underlie muscular degeneration. The asynchronous cycles of muscle fiber regeneration exacerbate muscle infiltration by macrophages and lymphocytes and their secretion of pro-inflammatory cytokines, leading to the replacement of myofibers with connective and adipose tissue, which becomes more evident with age progression [1]. Senescence, oxidative stress and inflammation—together with altered mitochondrial activity and proteostasis—are common features in aged organisms, and are the main pathogenetic mechanisms leading to muscle wasting in sarcopenia, which shares multiple features with DMD [2]. Moreover, detailed proteomic analysis of skeletal muscles from aged individuals highlighted a downregulation of proteins related to energetic metabolism and mitochondrial function and, conversely, an overexpression of signaling molecules regulating proteostasis, autophagy and innate/adaptive immunity [3].

Indeed, chronic inflammation observed in patients with DMD is often accompanied by increased oxidative stress in skeletal muscle, and both enhance the development of senescence. Further studies were conducted on dystrophic animal models and patients to elucidate its role in DMD progression. Sugihara and colleagues showed that inflammatory immune cells not only worsened oxidated stress but—interestingly—upregulated the secretion of pro-inflammatory cytokines specifically inducing senescence in satellite cells (SCs) and mesenchymal progenitor cells. This way, SC differentiation and proliferation were dramatically affected, as well as the capacity for self-renewal, possibly linking SC dysfunctions with DMD age-related defects in muscular functions [4]. According to these data, it was observed that modulation of clinical parameters and body composition of DMD patients becomes more evident with aging, as the increase of fat mass and decrease of lean mass as well as metabolic energy expenditure [5] were dependent on disease progression. 

In the last decade, given the role of the proteolytic system in regulating oxidative stress and proteostasis in aged muscle [6], several studies demonstrated that the inducible form of proteasome—immunoproteasome (IP)—is involved in these signaling pathways. A plethora of pro-inflammatory cytokines, such as tumor necrosis factor (TNF)-α and interferon (IFN)-γ, determine the transformation of the catalytically active subunits of the standard proteasome into inducible ones, leading to modulation of the peptide–bond cleavage capacity of the 20S core particle [7,8]. Other than regulating class-I ligand generation, IP coordinates antigen presentation, cytokine production, T cell differentiation and cytotoxic T cell response [9]. Interestingly, it was upregulated in response to stress and injury [10] and functioned as a regulator of skeletal muscle differentiation [7]. By analogy, in a denervation model of sarcopenia, Liu et al. described an upregulation of immunoproteasome content that, although not directly responsible of muscle wasting, might trigger signaling events ultimately enhancing the proteolytic pathways of the cell [11]. 

Since the majority of IP-dependent phenomena are commonly altered in DMD, we studied IP inhibition in 3-month-old (3m) mdx mice with ONX-0914—an IP inhibitor that recently entered clinical development phase I/II trials for patients with autoimmune-triggered inflammation—and observed a modification in the amount of regulatory (Tregs) and effector T cells and the expression of pro-inflammatory cues, modulating the fate of muscle-derived stem cells and their myogenic/adipogenic differentiative capacity. Consequently, the use of ONX-0914 in the context of DMD has been exploited with positive results in terms of fibrosis lowering, inflammatory component modulation, muscle mechanical behavior recovery, and muscle force improvement [12,13]—overall resulting in a slowdown of muscle wasting in mdx mice, DMD murine model.

Thanks to the lengthening of life expectancy according to valuable management of respiratory involvement, cardiac problems became the main cause of death for DMD patients. These individuals suffer from progressive ventricular dilation caused by myocyte loss and from enhanced apoptosis and fibrosis affecting the epicardium and the endocardium [14]. Since the signs of cardiomyopathy are evident in older mdx mice (9-month-old, 9m), we previously investigated the effects of ONX-0914 on 9m mdx cardiac pathology and observed an amelioration of inflammation, fibrosis and hemodynamic performance in the hearts, leading to slower progression of cardiac dysfunction. Of note, we also described a modulation of IP-dependent pathways into DMD patients’ cardiomyocytes [15]. 

It is now commonly accepted that immune cells, muscle fibers and associated cells operate in delicate balance to promote muscle regeneration following injury; this equilibrium is compromised in DMD pathology causing fibrosis, fat accumulation and sustained inflammation. Based on the knowledge that the above mentioned inflammatory and oxidative phenomena become more pronounced with aging, we expected that systemic beneficial effects of IP inhibition, such as preservation of healthy muscle mass, may also be evident in 9m aged mdx mice and not be limited to cardiac pathology. In the present study, we clearly demonstrate a positive modulation of inflammatory cells and metabolic pathways both in skeletal and cardiac tissues. These data suggest that immunoproteasome is a valuable target for positively modulating skeletal muscle functionality in old dystrophic mice and, intriguingly, it could constitute a feasible therapeutical option for counteracting skeletal muscle loss in DMD patients and, possibly, other subjects suffering from cancer-induced pathologies or multiple sclerosis.

## 2. Results 

### 2.1. Immunohistological Analysis of Skeletal Muscle Reveals Higher Percentage of Small Fibers and Metabolic Switch in Mdx Mice Treated with IP Inhibitor 

**Figure 1:**
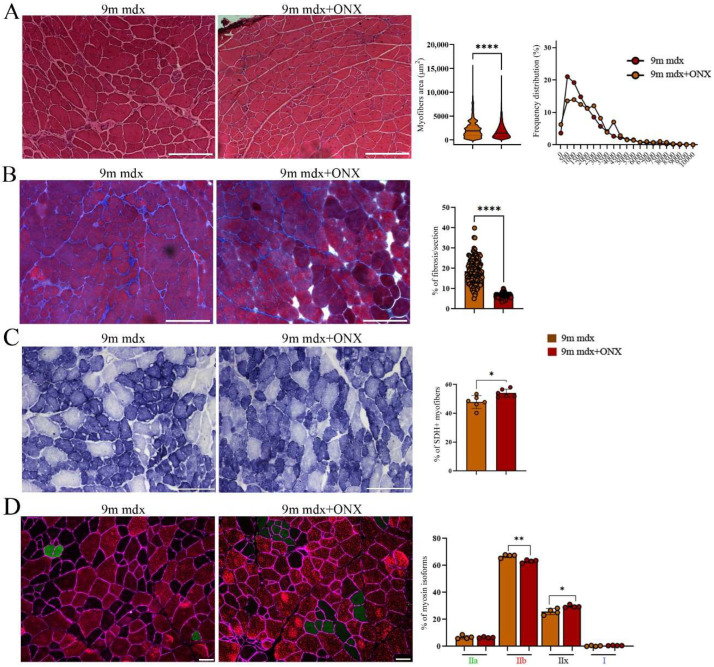
Histological and immunofluorescence analysis of tibialis anterior section of 9m mdx and 9m mdx+ONX mice. (**A**) Representative H&E staining and quantification of myofiber area and relative frequency of myofiber cross-sectional areas (CSA) expressed as frequency distribution of TA muscles of 9m mdx and 9m mdx+ONX mice *(n* = 10 images for animal group). Scale bar: 200 μm. (**B**) Quantification of fibrotic area in TA muscles of 9m mdx and 9m mdx+ONX mice (*n* = 10 images for animal group). (**C**) Representative SDH staining and quantification of percentage of SDH+ myofibers in TA of 9m mdx and 9m mdx+ONX mice (*n* = 3 for all groups). Scale bar: 200 μm. (**D**) Representative images of immunofluorescent staining showing distribution of myosin heavy chain (MyHC) isoforms (Type I, Type IIa, Type IIx and Type IIb) (*n* = 8 images were analyzed for each mouse). Graph portrays the percentage of myofibers expressing different MyHC isoforms. Scale bar: 50 μm. For morphometric analysis, images were quantified with Image J software. (* *p* < 0.05, ** *p* <0.01, **** *p* < 0.0001, with unpaired *t*-test).

Nine-month-old mdx mice were treated with ONX-0914 (9m mdx+ONX) for 4 weeks as previously described [15] and we evaluated how this treatment modulated skeletal muscle morphology. A significant downregulation of the myofibers area, associated with increased frequency of smaller myofibers, was observed in ONX-0914 treated mice (Figure 1A). As expected, this situation was accompanied by a decrease in fibrosis (Figure 1B). To assess the metabolic adaptations of skeletal muscle in aged mdx+ONX mice [16], we measured the mitochondrial enzyme succinate dehydrogenase (SDH) as indirect measurement of oxidative capacity [17]. TA muscle analysis of 9m mdx+ONX mice showed a significant increase in SDH+ fibers compared to untreated ones (Figure 1C). To assess whether the anti-inflammatory features exerted by ONX-0914 on skeletal muscle were associated with fiber type switch, we detected and quantified the immunoreactivity for adult myosin heavy chain isoforms (MyHC) through immunofluorescence. Interestingly, we found a high percentage of MyHC-IIx+ fibers, representing metabolically intermediate fibers with features common to both Type IIa and IIb, in 9m mdx+ONX mice, whereas untreated 9m mdx showed high percentage of glycolytic MHC-IIb fibers (Figure 1D). 

### 2.2. Serum Markers of Muscle Cell Damage Are Lowered by ONX-0914 Administration

**Figure 2:**
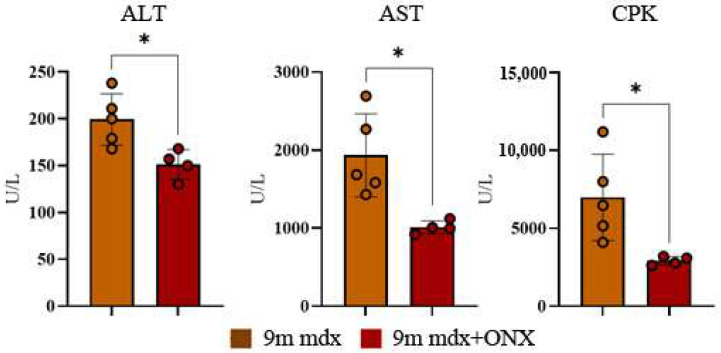
Serum ALT, AST and CPK levels in 9m mdx and 9m mdx+ONX mice. ALT, AST and CPK were measured in blood serum drawn from 9m mdx (*n* = 5) and 9m mdx+ONX mice (*n* = 4). Data are expressed as means ± SD of *n* = 4–5 9m mdx and 9m mdx+ONX mice (* *p* < 0.05, with unpaired *t*-test).

It is well known that in aged mice muscle atrophy is caused by an imbalance in proteostasis, often accompanied by muscle weakness. This way, to evaluate the effects of ONX-0914 on inflammation and possibly on muscular membrane integrity—the most common determinants of muscle fibers’ degeneration in aged dystrophic mice—we measured biochemical enzymes (ALT, AST and CPK) in blood serum. Accordingly, we found a significant downregulation of all these parameters in treated versus untreated mice (Figure 2).

### 2.3. ONX-0914 Modulates Skeletal Muscle and Spleen Immune Cell Population

**Figure 3:**
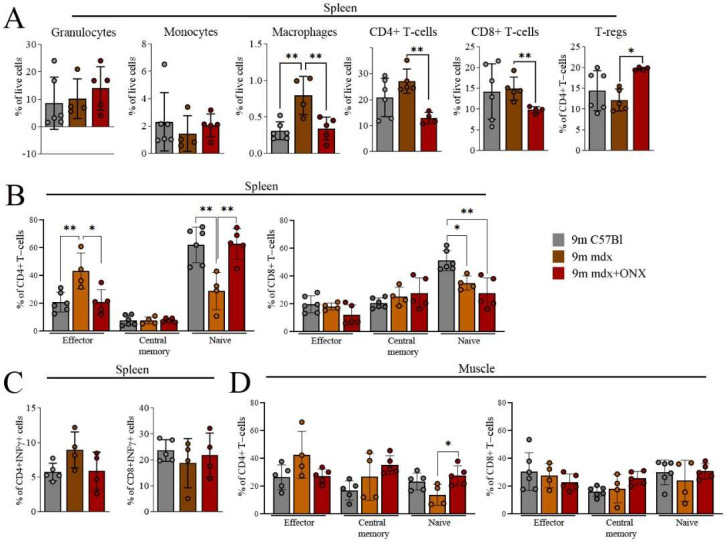
FACS analysis of immune cell population from spleen and skeletal muscles of 9m C57Bl, 9m mdx and 9m mdx+ONX mice. FACS analysis was performed on spleen and muscle homogenates from C57Bl, 9m mdx and 9m mdx+ONX mice. Panels for spleen show (**A**) percentages of granulocytes (Ly6g+), monocytes (Ly6c+), macrophages (F4/80+CD11b+), CD4+ and CD8+ T cells, and Tregs (FOXP3+ on CD4+ T-cell gate); (**B**) frequency of effector (CD62L−CD44+), central memory (CD62L+CD44+) and naïve (CD62L+CD44−) T cells within the CD4+ or CD8+ populations; (**C**) frequency of CD4+ and CD8+ IFNγ-producing cells. (**D**) Panel for muscle displays frequency of effector (CD62L−CD44+), central memory (CD62L+CD44+) and naïve (CD62L+CD44) T-cells within the CD4+ or CD8+ populations. Data are expressed as means ± SD of *n* = 2 independent experiments with *n* = 6 C57Bl mice and *n* = 4–5 9m mdx and 9m mdx+ONX mice (* *p* < 0.05, ** *p* < 0.01, with ordinary one-way ANOVA, Tukey’s multiple comparison test).

We analyzed the abundancy of inflammatory cell subpopulations in skeletal muscles and spleens of healthy (C57Bl) and ONX-treated and untreated mdx mice using flow cytometry. The increased proportion of splenic macrophages observed in old mdx mice was counteracted by ONX-0914 administration, with a reduction to amounts observed in C57Bl mice (Figure 3A and Supplementary Figure S1). A similar effect was observed for splenic CD4+ and CD8+ T lymphocytes. Of note, anti-inflammatory and pro-regenerative FoxP3+ Tregs were significantly increased in ONX-treated mice (Figure 3A). ONX-0914-mediated splenic immune cell modulation was confirmed by the increased proportion of naïve CD4+CD62L+CD44− cells and decreased effector memory CD4+CD62L−CD44+ and CD8+CD62L−CD44+ T cells compared to untreated 9m mdx mice (Figure 3B and Supplementary Figure S2), reaching a level similar to control mice. IFN-γ producing CD4+ and CD8+ T cells remained unaltered (Figure 3C). We did not find significant downregulation of effector cells among CD4+ and CD8+ subpopulations isolated from skeletal muscles, although a trend can be noticed, as shown by the upregulation of naïve CD4+CD62L+CD44− T cells in 9m mdx+ONX mice compared to untreated mice (Figure 3D and Supplementary Figure S3). Therefore, IP inhibition with ONX-0914 exerted a beneficial effect on T cells with inflammatory properties in aged mdx mice, as previously observed for younger mdx mice [12].

### 2.4. Proteomic Markers of Inflammation, Fibrosis and Oxidative Stress in Skeletal and Cardiac Muscle Are Modulated by ONX-0914 Administration

**Figure 4:**
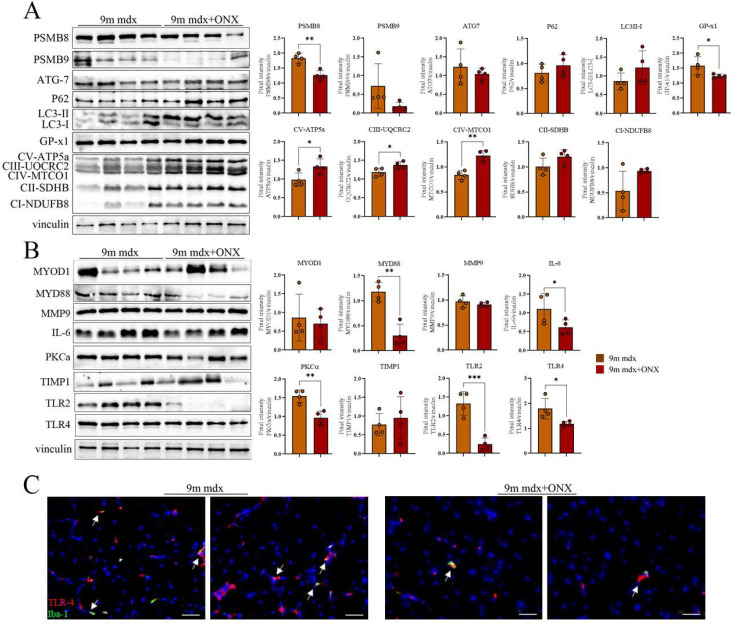
Anti-inflammatory and fibrotic effects of ONX-0194 on skeletal muscle of 9m mdx mice. Cropped images of representative WB showing the expression of proteins involved in (**A**) immunoproteasome, autophagy, oxidative phosphorylation, and (**B**) fibrosis and inflammation in skeletal muscles from 9m mdx mice treated with ONX-0914 versus untreated mice. Densitometric analyses of protein expression is shown as a ratio on vinculin. (**C**) Fluorescent staining of TLR-4 (in red) and Iba-1 (in green) while nuclei are in DAPI (blu) (scale bar: 50 μm) in TA of 9m mdx and 9m mdx+ONX. Macrophages expressing TLR-4 are evidenced by white arrows. Data are expressed as means ± SD of *n* = 3 independent experiments with *n* = 4 mice for animal groups (* *p* < 0.05, ** *p* < 0.01, *** *p* < 0.001, with unpaired *t*-test).

Next, we evaluated the effects of our treatment on fibrotic and inflammatory pathways in skeletal muscle via proteomic analysis. As expected, we observed a downregulation of the inducible PSMB8 and PSMB9 immunoproteasome subunits in treated mice (Figure 4A). In line with these evidences, we found a significant downregulation of GPx1, which is necessary for proper function of free radical metabolism [18]. Indeed, when pathologically modulated, it affects not only the resistance to pro-inflammatory oxidants but, importantly, the proliferation and apoptosis of myoblasts, muscular differentiation and quiescent satellite cell activity [19]. Conversely, we observed an upregulation of several components of the electron transport mitochondrial chain (OXPHOS complexes), possibly affecting metabolite homeostasis [20] and mitochondrial function and efficiency [21] (Figure 4A). Surprisingly, we did not observe significant modulation of autophagy. The amount of multiple inflammatory mediators was significantly diminished in treated mice. In particular, ONX-0914 treatment determined the downregulation of myeloid differentiation primary response 88 (MYD88), an adaptor protein that promotes the activation of several inflammatory pathways in response to signaling driven by toll-like receptors (such as TLR2 and TLR4, both downregulated in 9m mdx+ONX mice) and the interleukin-1 receptor [22] (Figure 4B). In addition, we found that ONX-0914 negatively modulated interleukin-6 (IL-6), the master regulator of the inflammatory machinery, and the protein kinase C (PKC)-α, which is known to modulate pathways involving NF-κB, NO and MAPKs, and to coordinate heart function and contraction/relaxation of myocardium in muscular dystrophies (Figure 4B). Intriguingly, since we found that reduced TLRs and MyD88 are normally expressed by macrophages, we extended our analysis evaluating the co-expression of Iba-1, a common macrophage marker, with TLR-4 and we clearly demonstrated that there was a downregulation of cells expressing TLR-4 in 9m mdx+ONX muscles (Figure 4C). 

**Figure 5:**
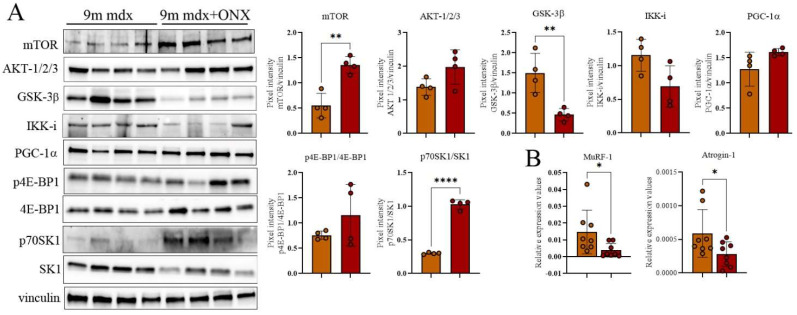
Evaluation of muscle wasting in 9m mdx and 9m mdx+ONX mice. Cropped images of representative WB showing (**A**) the expression of proteins involved muscle wasting as GSK-3β, IKK-i and PGC-1α; mTOR/AKT signaling and its most-common downstream targets 4E-BP1 and SK in skeletal muscles from 9m mdx mice treated with ONX-0914 versus untreated mice. Densitometric analyses of protein expression is shown as ratio on vinculin or ratio phosphorylated/total isoforms. (**B**) RT-qPCR analysis of *MuRF-1* and *atrogin* genes TA of 9m mdx and 9m mdx+ONX. Data are expressed as means ± SD of *n* = 3 independent experiments with *n* = 4 mice for animal groups (* *p* < 0.05, ** *p* < 0.01, **** *p* < 0.0001, with unpaired *t*-test).

To demonstrate further that ONX-0914 determined an amelioration of muscle wasting in 9m mdx mice, we followed the expression in WB of different proteins involved in this field. Taking into account the role of the Akt/mTOR pathway in regulating skeletal muscle growth and atrophy [23], we assessed that treated muscles significantly improved the expression of mTOR and also slightly increased AKT 1/2/3 (Figure 5A). Since it was shown that mTOR deficiency not only impaired the metabolic pathways of muscles but imbalanced the phosphorylation of different downstream proteins [24], we described the ratio p70SK1/SK1 as dramatically upregulated (Figure 5A). Indeed, we showed a significant decrease of GSK-3β, whose inhibition in skeletal muscle was associated to amelioration of fatigue and force [25] and rescue of regeneration in atrophic muscles [26] (Figure 5A). RT-qPCR analysis confirmed the downregulation in 9m mdx+ONX muscles of genes closely associated with wasting and atrophy, as *Muscle RING-finger protein-1 (MuRF1)* and *atrogin* (Figure 5B).

**Figure 6:**
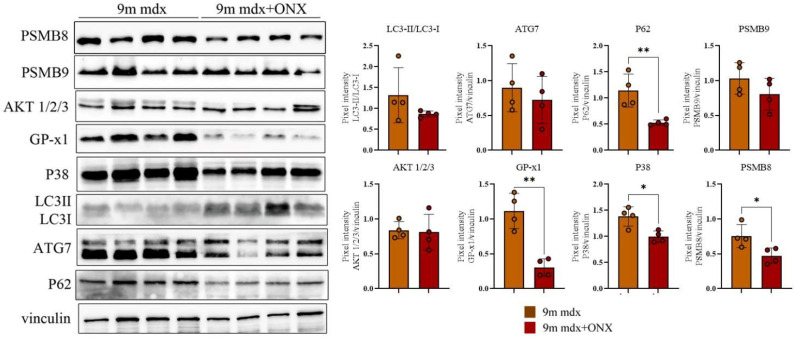
Proteomic analysis of whole cardiac muscle lysates from 9m mdx and 9m mdx+ONX mice. Cropped images of representative WB showing the expression of proteins involved in autophagy, fibrosis and inflammation in cardiac tissues from 9m mdx mice treated with ONX-0914 versus untreated mice. Densitometric analyses of protein expression is shown as ratio on vinculin. Data are expressed as means ± SD of *n* = 3 independent experiments with *n* = 4 mice for animal groups (* *p* < 0.05, ** *p* < 0.01, with unpaired *t*-test).

In the heart, we observed a significant downregulation of P62, GP × 1—whose effects on cardiac hypertrophy [27] and mitochondrial efficiency [28] are well recognized—and p38 MAPK, which fosters heart failure by promoting pro-inflammatory cytokine production and extracellular matrix remodeling [29] (Figure 6). 

### 2.5. In Diaphragms of Aged mdx Mice

**Figure 7:**
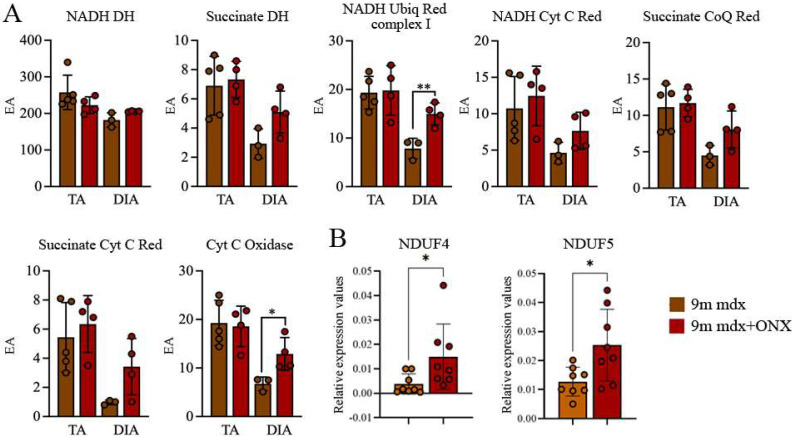
Mitochondrial respiratory chain enzymatic activity in TA and DIA of 9m mdx and 9m mdx+ONX mice. (**A**) Effects of ONX-0914 treatment on the activity of muscle (TA and DIA) respiratory chain enzymes in 9m mdx and 9m mdx+ONX mice (EA: enzymatic activity; NADH DH: NADH dehydrogenase; Succinate DH: succinate dehydrogenase; NADH Ubiq Red complex 1: NADH ubiquinone reductase complex 1; NADH Cyt C Red: NADH cytochrome C reductase; Succinate CoQ Red: succinate CoQ reductase; Succinate Cyt C Red: succinate cytochrome C reductase; Cyt C oxidase: cytochrome C oxidase). (**B**) RT-qPCR analysis of *NDUF4* and *NDUF5* in DIA of 9m mdx and 9m mdx+ONX mice. Data are expressed as means ± SD of *n* = 2 independent experiments with *n* = 4–5 9m mdx and 9m mdx+ONX mice (* *p* < 0.05, ** *p* < 0.01, with unpaired *t*-test). ONX-0914 upregulates NADH ubiquinone 1 reductase and of cytochrome oxidase.

It was suggested that the dysfunction of muscular energetic systems in DMD animal models affects correct ATP production and negatively modulates the flux of calcium ions, leading to dramatic fiber regeneration issues [30]. Consistently, Rybalka et al. demonstrated that mitochondria isolated from mdx tibialis anterior (TA) and diaphragm shared failures in Complex I-mediated mitochondrial ATP production rates [31]. Accordingly, we investigated the mitochondrial enzymes and did not detect differences in TA; however, we found an upregulation of NADH ubiquinone 1 reductase and of cytochrome oxidase in diaphragms of 9m mdx+ONX mice (Figure 7A). To confirm this evidence, we evaluated the expression of a component of the cytochrome c oxidase (NADH-Ubiquinone oxidoreductase MLRQ subunit, NDUFA4) and of the Complex I of the mitochondrial respiratory chain (NADH-Ubiquinone oxidoreductase 13 KDa-B subunit, NDUFA5) and we demonstrated that these genes were overexpressed in treated 9m mdx mice (Figure 7B). As suggested by Duan [32], ONX-0914 increases the amount of these enzymes and favors the rescue of the diaphragm’s pathological phenotype, improving metabolism and oxidative stress in aged 9m mdx mice. 

## 3. Discussion

Regardless of its origin, severe loss of muscle mass or sarcopenia leads to a significant decline in quality of life in terms of decreased independence and susceptibility to diseases. However, the mechanisms behind this pathological condition are not yet fully understood and are likely multifactorial. As in sarcopenia, DMD inflammatory cells exacerbate muscle wasting and protein oxidation, and represent the primary feature of dystrophic muscle damage [33,34], both in murine animal models [35] and DMD patients [36]. With aging and cell senescence being fundamental causes of muscle loss, we hypothesized that 9-month-old mdx mice can be considered a suitable murine model for investigating the events triggering muscle wasting and, consequently, as allowing for clinical improvements on this debilitating condition. Immunoproteasome activity is enhanced in the dystrophic context: IP regulates the expression of proteins involved in the mTOR/Akt pathway and in many pathological features triggered in frailty individuals. The rapamycin, an inhibitor of mTOR, reduces the expression of IP subunits [37], while mTORC1 coordinates protein synthesis and IP formation to prevent accumulation of protein stress [38]. IP is involved in NF-kB-dependent inflammation [12] and coordinating oxidative stress-adaptive proteolytic complexes [39,40]. Accordingly, dysregulated IP activity—whether it be over- or underactive—has been linked to abnormal muscle mass maintenance and muscle atrophy, as demonstrated by research on several models of muscle wasting and denervation [11], muscular dystrophy [41], inflammatory myopathies. Intriguingly, taking into consideration that defects in proteostasis are involved in muscle integrity [42], Fletcher et al. showed that detrimental activity of proteasome and IP can determine reduced muscle mass and strength in obese subjects [43]. However, IP’s role in regulation of myoblast differentiation is still to be elucidated as previous findings support the idea that IP inhibition impairs C2C12 differentiation in vitro [7]. 

Other studies demonstrated that IP expression increased according to enhanced oxidative stress and aging [44], probably as a compensative effect since IP preferentially degraded oxidized proteins with greater activity and selectivity related to that of the 20S proteasome [40]. Indeed, Pickering et al. assessed that IP (together with proteasome) function modulation could contribute to aging and longevity determination, not only favoring the turnover of damaged proteins but also increasing the MHC Class I cell surface antigen presentation and consequently the activation of the immune system [45]. 

Overall, inhibition of IP has shown promising results in countering muscle wasting in young (3-month-old) mdx mice. Here, we further expanded the knowledge in this field, highlighting the efficacy of ONX-0914 to reduce the overexpression of IP and to ameliorate the already established and advanced pathological phenotype of 9m mdx mice, by modulating the main pathways that cause sarcopenia.

The amelioration of the pathological phenotype is initially evident in the global conditions of the skeletal muscle at histological level, where the marked decrease of fibrosis and the smaller cross-sectional area of the fibers suggest a more efficient regenerative processes occurring in the tissues of treated mice, as supported by biochemical markers of muscle cell death. Therefore, it appears that IP inhibition dampens the uncontrolled degeneration/regeneration cycle observed in muscular dystrophy that ultimately leads to exhaustion and muscle atrophy.

Flow cytometry analysis of inflammatory populations in the spleen and muscles is indicative of a shift from a pro-inflammatory environment, sustained by both innate and adaptive immune cells, namely T-cells and macrophages, to a more anti-inflammatory one in treated mice. In particular, we found that ONX-0914 treatment increased the proliferation of Tregs and naïve T-cells while diminishing the amount of CD4+ effector T-cells in accordance with their opposite role in aging and muscle wasting [46,47,48].

Consistent findings were observed using Western blot analysis, leading to downregulation of inflammatory markers such as MyD88, TLRs, IL-1R, IL-6 and PKCα. Intriguingly, we showed that ONX-0914 dampened the amount of macrophages expressing TLR-4, suggesting that the reduction of pro-inflammatory cues could be partly ascribed to inhibition of these cells. A major evidence of our study was, indeed, the upregulation of mTOR-dependent signaling. In fact, we found that ONX-0914 increased the expression of mTOR and mTOR targets such as 4E-BP1 and—especially—SK1. In accordance with the metabolic and morphological rescue described in treated muscles, we assessed that other proteins acting synergically with mTOR such as GSK-3β and PGC-1α were differentially expressed, enhancing regulation of protein turnover and muscle oxidative capacity and reducing sarcopenia and muscle fiber growth as previously described [26,49,50,51]. 

IP attenuates myocardial destruction and preserves cell vitality in heart tissue inflammation [52] by regulating ERK1/2 and p38 MAPKs in a PTX-3-dependent mechanism [53]. In murine models and in fibroblasts from adrenoleukodystrophy patients’ proteasome and IP did not function properly due to the accumulation of oxidized proteins [54], causing the uncontrolled ubiquitination of mitochondrial proteins [55,56,57]. Antioxidant treatment prevents IP translocation into mitochondria, suggesting IP involvement in mitochondrial quality control [54]. A recent study showed that mitochondrial dysfunction in aged mouse lungs determined downregulation of oxidative phosphorylation and, on the contrary, a significant increase in immune response pathways dependent on IP function [58]. In line with these findings, we observed relevant modifications at the metabolic level following ONX-0914 administration, as indicated by the upregulation of SDH+ fibers and modulation of myofiber type frequency and distribution. Hence, ONX-0914 administration buffers the oxidative milieu commonly observed in DMD pathology, possibly by stabilizing mitochondrial activity. 

Although administration of ONX-0914 to 9m mdx mice constitutes a late intervention is unlikely to lead to rescue of the healthy phenotype, a partial improvement as the one observed in our study is highly desirable. Muscle atrophy strongly impacts the quality of life in patients with DMD, leading to weakness, fatigability and heavy restriction in daily activities. The total ablation of the immune component—as occurs following steroid treatment, although resolving inflammation, results in poor muscular regeneration and does not impact positively on muscle wasting. Here, we demonstrated how ONX-0914 influenced different immune players and mitochondrial stability, exerting its effect on several pro-inflammatory pathways and the muscular metabolism. Palliative interventions aiming at the maintenance of muscle mass are crucial in the overall management of dystrophic patients and, most importantly, in a globally aging population. ONX-0914 is a promising molecule, with relatively low side-effects, that could expand the contemporary therapeutic arsenal available for treating patients with dysregulated muscle mass. 

## 4. Materials and Methods

### 4.1. Ethical Statement 

The research procedures described were approved by the ethics committee of the University of Milan (CR937-G). This study was performed in accordance with the International Conference on Harmonisation of Good Clinical Practice guidelines, the Declaration of Helsinki (2008) and the European Directive 2001/20/EC. Procedures involving living animals were approved by local ethics committees, conforming to Italian law (D.L.vo 116/92 and subsequent additions). This work was authorized by the Ministry of Health and Local University of Milan Committee with the protocol authorization numbers 10/10-2009/2010 and 6/13-2012/2013. Nine-month-old normal (9m C57Bl) and dystrophic (9m mdx) mice were provided by Charles River (Calco, Lecco, Italy) and caged in comfort and safety, in a controlled ambience (12 h light, 12 h dark) at a temperature between 21 °C and 24 °C. The mice had free access to clean water and food. The immunoproteasome inhibitor ONX-0914 (Clini Sciences—Nanterre, France, 6 mg/Kg) was injected intraperitoneally into 9m mdx mice (9m mdx+ONX) for four weeks (two injections per week, *n* = 10). Untreated age-matched mdx mice were used as control. Mice were sacrificed by cervical dislocation according to Italian law.

### 4.2. Serum Analysis

CPK, ALT and AST analyses were performed on serum samples of 9m mdx and 9m mdx+ONX mice CPK/ALT/AST kit (Cobas, Roche, Basilea, Switzerland), according to manufacturer’s instructions.

### 4.3. Flow Cytometry Analysis

Muscles and spleen were removed from mice to determine the amount of different immune subpopulations using flow cytometry analysis. Muscles were excised and extensively washed in PBS to remove blood contaminants [59], cut in small pieces and digested for 1 h with liberase 0.2 mg/mL (Life Technologies, CA, USA). Digested muscle tissue after filtration through a 70-μm filter was placed on Histopaque 1,077 gradient. The gradient was centrifuged at 400× *g* for 45 min, and the cells at the interface were harvested. Red blood cells were lysed by adding 2 mL of ACK Lysis buffer, purchased from ThermoFisher Scientific (Life Technologies, CA, USA). The cells were washed two times with PBS, counted and then used for flow cytometry analysis. Cell suspensions from muscles and spleen were labelled using the following antibodies for muscle (CD45 PerCp-Cy5.5; CD4 AF405; CD8 APC-Cy7; CD44 FITC; CD62L PE) and for spleen (CD4 AF405; CD8 PerCp-Cy5.5; CD44 FITC; CD62L PE. Foxp3 AF488; CD25 APC; Ly-6C AF488; Ly-6G APC; F4/80 Pe-Cy7). Antibodies were purchased from eBioscience (Life Technologies, CA, USA) and BioLegend (San Diego, CA, USA). Labelled cell suspensions were acquired by FACS Canto II (BD Bioscience, NJ, USA) and data analyzed with FlowJo 9.9.6 software, Ashland, OR, USA.

### 4.4. WB Analysis

Total proteins were obtained from skeletal muscles and hearts isolated from 9m mdx and 9m mdx+ONX mice. Samples were resolved on polyacrylamide gels (ranging from 6% to 12%), transferred to nitrocellulose membranes (Bio-Rad Laboratories, CA, USA) and incubated overnight with following antibodies: vinculin (1:600, MA5-11690, Life Technologies, CA, USA); p38 (1:500, E-AB-32460, Elabscience, Houston, USA); PSMB8 (1:500, Proteasome 20S LMP7, ab3329, Abcam, Cambrdige, UK); PSMB9 (1:500; Proteasome 20S LMP2 (EPR13785) ab184172, Abcam, Cambrdige, UK); GPx1 (1:500, ab22604, Abcam, Cambrdige, UK); MyoD (1:500, 554130, BD, NJ, USA); IL-6 (1:500, sc-57315, Santa Cruz Biotechnology, Dallas, USA); TLR4 (1:500, sc-293072, Santa Cruz Biotechnology, Dallas, TX, USA); TIMP1 (1:500, ab86482, Abcam, Cambrdige, UK); TLR2 (1500, orb229137, Biorbyt, Cambridge, UK); PKCα (1:600, 610108, BD, NJ, USA); MMP9 (1:500, ab38898, Abcam, Cambrdige, UK); ATG7 (1:500, sab4200304, Sigma-Aldrich, St. Louis, USA); LC3B (1:500, L7543, Sigma-Aldrich, St. Louis, USA); OXPHOS (1:500, MS604-300, Abcam, Cambrdige, UK); P62 (1:500, P0067, Sigma-Aldrich, St. Louis, USA); MYD88 (1:500, 23230-1-AP, Proteintech, Rosemont, USA); AKT1/2/3 (1:500, ab126811, Abcam, Cambrdige, UK); IKK-i (1:600, sc-10760, Santa Cruz Biotechnology, Dallas, TX, USA) p70 S6 Kinase 1 (1:600, #9202, Cell Signaling Technology, Danvers, MA, USA); Phospho-p70 S6 Kinase 1 (1:600. #9205, Cell Signaling Technology, Danvers, MA, USA); 4E-BP1 (53H11) (1:600, #9644, Cell Signaling Technology, Danvers, MA, USA); Phospho-4E-BP1 (1:600. #2855, Cell Signaling Technology, Danvers, USA); mTOR (7C10) (1:600, #2983, Cell Signaling Technology, Danvers, MA, USA). Proteins of interests were detected with peroxidase conjugated secondary antibodies (Agilent Technologies, CA, USA) and developed by ECL (Amersham Biosciences, Amersham, UK). Bands were quantitated in ImageJ software. 

### 4.5. Histological and Immunofluorescence Analysis of Muscle Sections

Histological and immunofluorescence analyses were performed on murine tissue sections. Muscular biopsies were collected from 9m C57Bl, 9m mdx and 9m mdx+ONX, frozen in liquid-nitrogen-cooled isopentane and cut on a cryostat into 10 µm slices. H&E staining was performed as in [60] to evaluate the morphology of muscles. SDH staining was performed by incubating slides in SDH solution for 1 h at 37 °C. Slides were later exposed to a scale of acetone solution (30%, 60%, 90%, 60%, 30%), each for 10 s, and dehydrated in a scale of ethanol solutions (80%, 90%, 100%), each for 45–60 s. Finally, slides were exposed to 100% xylene for 30 s and mounted with DPX reagent and coverslips. Evaluation of fibrosis level was performed by Azan–Mallory staining. A 1:1 solution of Weigert Ferric Hematoxylin Solution A and B (Bio-Optica S.p.A., Milan, Italy) was applied to tissue sections for 10 min. Slices were then exposed to a 100% picric acid solution for 2 min. Fucsin Ponceau Masson 100% (Bio-Optica S.p.A., Milan, Italy) combined with 1% glacial acetic acid (Carlo Erba, Milan, Italy) was applied for 7 min. Slides were later put in a 100% phosphomolybdic acid (Bio-Optica) solution for 5 min and 15% aniline blue (Bio-Optica S.p.A., Milan, Italy) for 1 min. Three washings in distilled water were performed between each incubation step. After incubation in 1% glacial acetic acid for 1 min, sections were exposed for 30 s to a 100% ethanol solution for dehydration, and finally to 100% xylene for 1 min, before mounting with DPX and coverslips. Immunofluorescence staining for identification of muscle fibers type was performed as follows. Sections were fixed with ethanol and acetone (1:1) for 3 min at RT, followed by 3 washings in PBS 1×, 5 min each. Antigen retrieval was performed by immersion in sodium citrate buffer (10 mM) at pH 6 at 100° for 25 min, followed by a 15 min cooling period and 3 washings with 0.05% Tween in PBS1X, 3 min each. Sections were incubated for 1 h with a blocking solution of 5% goat serum in PBS 1X at RT, and then incubated O/N at 4 °C with primary antibody, diluted in blocking solution: myosin heavy chain Type I BA-D5 (1:50, 115-675-207, DSHB, Iowa City, USA), myosin heavy chain Type IIa sc-71 (1:50, 115-545-205, DSHB, Iowa City, USA), myosin heavy chain Type IIb BF-F3 (1:50, 115-585-075, DSHB, Iowa City, USA). After 3 PBS 1× washes of 5 min, goat anti-mouse IgG 488 was diluted 1:100 in PBS 1X and added for 40 min at 37 °C to sections. After PBS 1X washing, goat anti-mouse IgG 421 and goat anti-mouse IgM 594 were diluted 1:100 in PBS 1X and added for 40 min at 37 °C to sections. Sections were washed with PBS 1X and incubated with laminin primary antibody (1:200, L9393, Sigma-Aldrich, St. Louis, USA) at RT for 2 h. After PBS 1X washing, goat anti-rabbit IgG 647 was diluted 1:100 in PBS 1X and added at 37 °C for 40 min to sections. Slides were mounted with PBS 1X-Glycerol (Sigma-Aldrich, St. Louis, MO, USA) at 1:1 ratio and coverslips. The stained sections were observed with a laser microdissection microscope and images were acquired by using the Leica Laser Microdissection. Densitometric analyses, manual or automatic counting were performed using ImageJ Software (imagej.nih.gov/ij/index.html, version 1.52t). Quantification was performed via ImageJ Software. Threshold color plug-in of ImageJ Software was used to quantify the amount of fibrosis in Azan–Mallory staining. Data were analyzed by GraphPad Prism^TM^ and expressed as means ± SD. 

For immunofluorescence experiments, sections were brought at RT from −80 °C and fixed with 4% PFA (Life Technologies, CA, USA) for 10 min at RT, followed by 3 washings in PBS 1X, 5 min each. PBS 1X + 0,01% Triton X-100 (Sigma-Aldrich, St. Louis, MO, USA) for 20 min at RT was used to permeabilize tissues. Sections were then incubated with a blocking solution of 10% donkey serum in PBS 1X, for 1 h at RT. Sections were then incubated overnight at 4 °C with TLR4 (1:50, sc-293072, Santa Cruz Biotechnology, Dallas, Tx, USA) and Iba-1 (1:50, e404w, Cell Signaling, Danvers, USA) primary antibody, diluted in blocking solution. After 3 PBS 1X washes of 5 min, donkey anti-rabbit secondary antibody 488 Plus (1:400, A32790, Life Technologies, CA, USA) was diluted in PBS 1X and added for 1h at room temperature to sections. After PBS 1X washing, donkey anti-mouse secondary antibody 594 Plus (1:400, A32744, Life Technologies, CA, USA) was diluted in PBS 1X and added for 1h at room temperature to sections. Nuclei were counterstained with DAPI (Molecular Probes, Life Technologies, CA, USA) for 5 min at RT. Slides were mounted with PBS 1X-Glycerol (Sigma-Aldrich, St. Louis, MO, USA) at 1:1 ratio and coverslips. Images were acquired with a Leica DMi8 fluorescence microscope.

### 4.6. Quantitative (RT-qPCR) Experiments 

Total RNA was extracted from DIA of 9m and 9m+ONX mdx mice and cDNA generated using the Reverse Transcriptase Kit (Life Technologies, CA, USA). The threshold cycles (Ct) of target genes were normalized against the housekeeping gene, β-actin. The expression of genes was quantified by means SYBR-Green method. The primers used are the following: β-actin-F GGCTGTATTCCCCTCCATCG and β-actin-R CCAGTTGGTAACAATGCCATGT; atrogin1-F CTTTCAACAGACTGGACTTCTCGA and atrogin1-R CAGCTCCAACAGCTCTACTACGT; MuRF-1-F GAGAACCTGGAGAAGCAGCT and MuRF1-R CCGCGGTTGGTCCAGTAG. The primers for NDUF4 were the same as published in [61] and for NDUF5 in [62].

### 4.7. Mitochondrial Enzymes 

We collected DIA and TA of 9m mdx and 9m mdx+ONX and we prepared these samples for the analysis of mitochondrial respiratory chain enzyme and citrate synthase activities as described in detail in [63]. The specific activity of each complex was normalized to that of citrate synthase.

### 4.8. Statistics

To determine significance when comparing multiple groups’ means, we used ordinary one-way ANOVA, with Tukey’s multiple comparison test; a two-way Student’s *t*-test was used to compare two groups assuming equal variances. The difference among groups was considered significant as follows: * at *p* < 0.05; ** at *p* < 0.01; *** at *p* < 0.001; **** at *p* < 0.0001. 

## Data Availability

All data are available in the main text.

## Supplementary Materials

The following supporting information can be downloaded at: https://www.mdpi.com/article/10.3390/ijms232314657/s1.
