# Supplementary material for: Immunoproteasome Inhibition Ameliorates Aged Dystrophic Mouse Muscle Environment
Source: Int J Mol Sci. 2022 Nov 24;23(23):14657. doi: 10.3390/ijms232314657 (PMC9739773; doi:10.3390/ijms232314657)
Supplement: Supplementary file 1 [file ijms-23-14657-s001.zip › ijms-1998051-supplementary.pdf]

Supplementary Figure S1

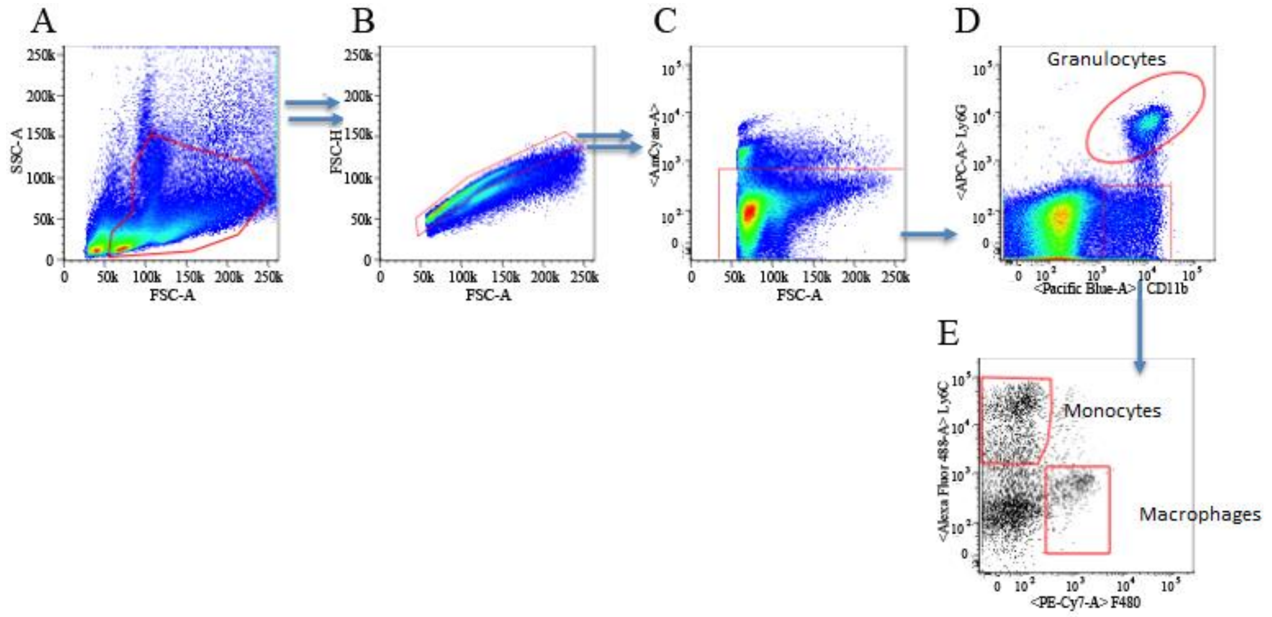

Figure S1. Flow cytometry gating strategy. Total splenocytes were first gated on a forward scatter (FS)/side scatter (SS) plot **(A)** and then a single cell gating (FSC-A x FSC-H) was performed to eliminate “doublets”, or cells stuck together **(B)**. Live/dead Aqua marker was used to identify live cells (Aqua negative cells) **(C)**. Ly-6G and CD11b markers were used to define the double positive population of granulocytes **(D)**. Ly-6C expression within the gate of CD11b+ Ly-6G $^{-}$ cells was used to identify monocytes, whereas CD11b x F4/80 gating was used to select macrophages **(E)**. Data were analyzed using FlowJo software, and population frequencies expressed as percent of live cell population.

Supplementary Figure S2

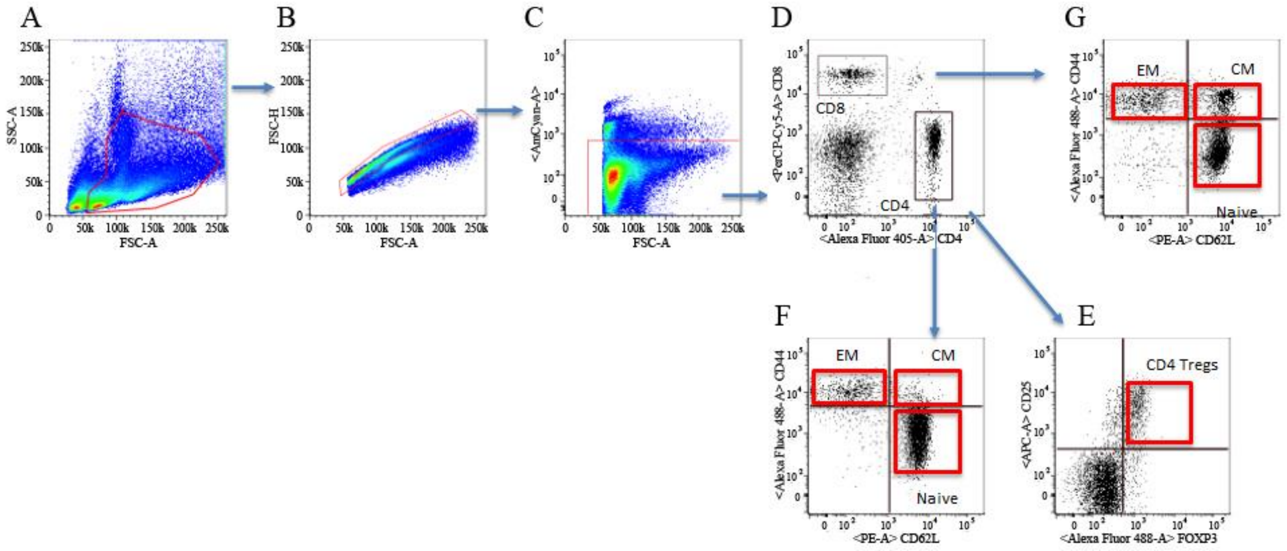

Figure S2. Flow cytometry gating strategy. Total splenocytes were first gated on a forward scatter (FS)/side scatter (SS) plot **(A)** and then a single cell gating (FSC-A x FSC-H) was performed to eliminate “doublets”, or cells stuck together **(B)**. Live/dead Aqua marker was used to identify live cells (Aqua negative cells) **(C)**. Cells were then gated on the CD4+ or CD8+ population **(D)**. These were further gated for the subsets of interest, namely, CD4+FOXP3+CD25+ regulatory T cells **(E)**, CD62L+CD44⁻ naive CD4+ or CD8+ cells, CD62L+CD44+ central memory CD4+ or CD8+ cells, and CD62L⁻CD44+ effector CD4+ or CD8+ cells **(F-G)**. Data were analyzed using FlowJo software, and sub-population frequencies expressed as percent of the CD4 or CD8 parent population.

Supplementary Figure S3

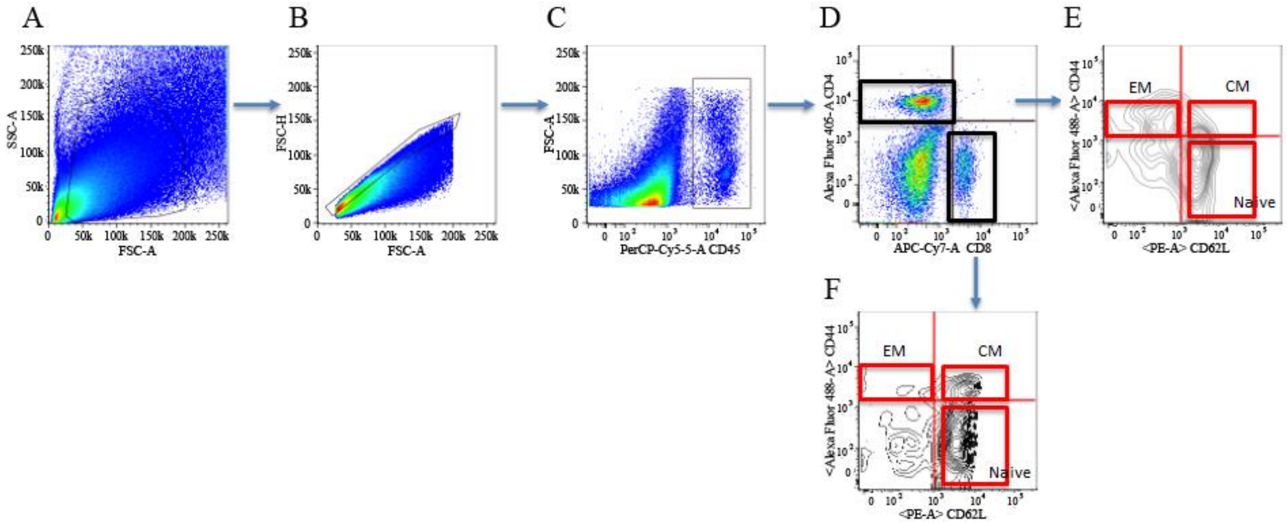

Figure S3. Flow cytometry gating strategy. Muscle cell suspensions, obtained as described in Methods were first gated on a forward scatter (FS)/side scatter (SS) plot **(A)** and then a single cell gating (FSC-A x FSC-H) was performed to eliminate "doublets", or cells stuck together **(B)**. CD45 marker was used to identify hematopoietic cells **(C)**. Cells were then gated on the CD4+ or CD8+ population **(D)**. These were further gated for the subsets of interest, namely, CD62L+ CD44– naive CD4+ or CD8+ cells, CD62L+ CD44+ central memory CD4+ or CD8+ cells, and CD62L–CD44+ effector CD4+ or CD8+ cells **(E-F)**. Data were analyzed using FlowJo software, and T cell frequencies were expressed as percent of the CD45 population. Lymphocyte subsets frequencies were expressed as percent of the CD4 or CD8 parent population.
